# An Open-Source Real-Time Motion Correction Plug-In for Single-Photon Calcium Imaging of Head-Mounted Microscopy

**DOI:** 10.3389/fncir.2022.891825

**Published:** 2022-06-24

**Authors:** Mingkang Li, Changhao Liu, Xin Cui, Hayoung Jung, Heecheon You, Linqing Feng, Shaomin Zhang

**Affiliations:** ^1^Key Laboratory of Biomedical Engineering of Education Ministry, Zhejiang Provincial Key Laboratory of Cardio-Cerebral Vascular Detection Technology and Medicinal Effectiveness Appraisal, Department of Biomedical Engineering, School of Biomedical Engineering and Instrument Science, Zhejiang University, Hangzhou, China; ^2^Qiushi Academy for Advanced Studies, Zhejiang University, Hangzhou, China; ^3^Department of Industrial and Management Engineering, Pohang University of Science and Technology, Pohang, South Korea; ^4^Research Institute of Artificial Intelligence, Zhejiang Lab, Hangzhou, China

**Keywords:** calcium imaging, motion correction, single-photon microscopy, real-time, open-source

## Abstract

Single-photon-based head-mounted microscopy is widely used to record the brain activities of freely-moving animals. However, during data acquisition, the free movement of animals will cause shaking in the field of view, which deteriorates subsequent neural signal analyses. Existing motion correction methods applied to calcium imaging data either focus on offline analyses or lack sufficient accuracy in real-time processing for single-photon data. In this study, we proposed an open-source real-time motion correction (RTMC) plug-in for single-photon calcium imaging data acquisition. The RTMC plug-in is a real-time subpixel registration algorithm that can run GPUs in UCLA Miniscope data acquisition software. When used with the UCLA Miniscope, the RTMC algorithm satisfies real-time processing requirements in terms of speed, memory, and accuracy. We tested the RTMC algorithm by extending a manual neuron labeling function to extract calcium signals in a real experimental setting. The results demonstrated that the neural calcium dynamics and calcium events can be restored with high accuracy from the calcium data that were collected by the UCLA Miniscope system embedded with our RTMC plug-in. Our method could become an essential component in brain science research, where real-time brain activity is needed for closed-loop experiments.

## 1. Introduction

In neuroscience research, the efficient recording and decoding of neural signals is essential. Although multielectrode extracellular recordings can continuously record hundreds of neurons (Buzsáki, 2004), the spatial relationships between the recorded samples cannot be captured (Gobel and Helmchen, 2007). Of the newly developed recording techniques, optical recording methods provide high-resolution image features and can be used with awake animals (Denk et al., 1990; Garaschuk et al., 2006). Among them, head-mounted microscopy (Ghosh et al., 2011)-based single-photon calcium imaging has been widely adopted as a tool for observing large neural populations in free-moving mice to explore neural coding mechanisms, such as how the anterior cingulate cortex mediates effort-based decisions (Hart et al., 2020), how the brain links memories across time (Cai et al., 2016) and how spatial coding breaks down in epilepsy (Shuman et al., 2020). However, when used with freely-moving mice, the activities of the animals can induce shaking in the imaging field of view (FOV), interfering with subsequent analyses of neuronal activity. Most current solutions to this issue involve postprocessing the acquired data with offline processing algorithms or toolboxes. For example, MIN1PIPE (Lu et al., 2018) is a single-photon-based calcium imaging signal extraction pipeline with high motion correction precision. This method scores the overall data, then divides the data into several groups for motion correction. As a result, parallel processing can be applied to multiple sections. This hierarchical approach can reduce the required time due to the balanced assignment of processing tasks. However, the motion correction module in MIN1PIPE still consumes most of the processing time and therefore cannot be used for real-time analysis. Other postprocessing toolboxes, such as CAVE (Tegtmeier et al., 2018), Suite2p (Pachitariu et al., 2017) and EZcalcium (Cantu et al., 2020), also include motion correction modules. Similar to MIN1PIPE, these toolboxes can only be used for *post hoc* analysis and cannot be used in closed-loop experiments that require real-time processing of the video stream during head-mounted microscopy.

The first requirement of real-time processing is that the flow of the algorithm satisfies the requirements of online processing. NoRMCorre (Pnevmatikakis and Giovannucci, 2017) is an online nonrigid subpixel registration method. It divides the imaging field into several overlapping patches and calculates the displacement of each patch. However, this method requires that all patches contain some salient image features after segmentation. In addition, because of the translation estimates for the patches and the overlap regions, the computation cost is higher than that of the rigid method; thus, its speed cannot meet the requirements of real-time processing. Although NoRMCorre also provides a rigid version for online processing, its speed is limited to real-time processing for small images or low frame rates. In terms of speed, Mitani (Mitani and Komiyama, 2018) provided a real-time motion correction tool for two-photon calcium imaging data. This was the first reported real-time calcium data processing pipeline to be used in closed-loop experiments. This method first shrinks the input frames, then registers them with the OpenCV template matching method before amplifying the detected translation to the original scale as the true translation. This method can process a 512 × 512 × 1,000 movie in less than 3 s. However, its accuracy on single-photon calcium imaging data is less reliable, most likely due to light scattering at the focal plane (Grienberger and Konnerth, 2012) and the fact that it includes fewer spatial features than the two-photon imaging data. Therefore, no algorithm or software can meet the requirements for acquiring single-photon calcium imaging videos without movement artifacts in real time.

To address this issue, we propose a real-time motion correction (RTMC) plug-in for single-photon calcium imaging of head-mounted microscopy that focuses on rigid translation correction. The RTMC algorithm can be embedded in UCLA Miniscope data acquisition (DAQ) software and used to directly capture stable single-photon calcium imaging videos on a microscope by running an online registration algorithm on a GPU. We show that the RTMC algorithm can correct a single frame with high accuracy in approximately 15 ms, and that it can be used in real-time experiments with behaving mice.

## 2. Materials and Methods

### 2.1. Algorithm Description

**Figure 1:**
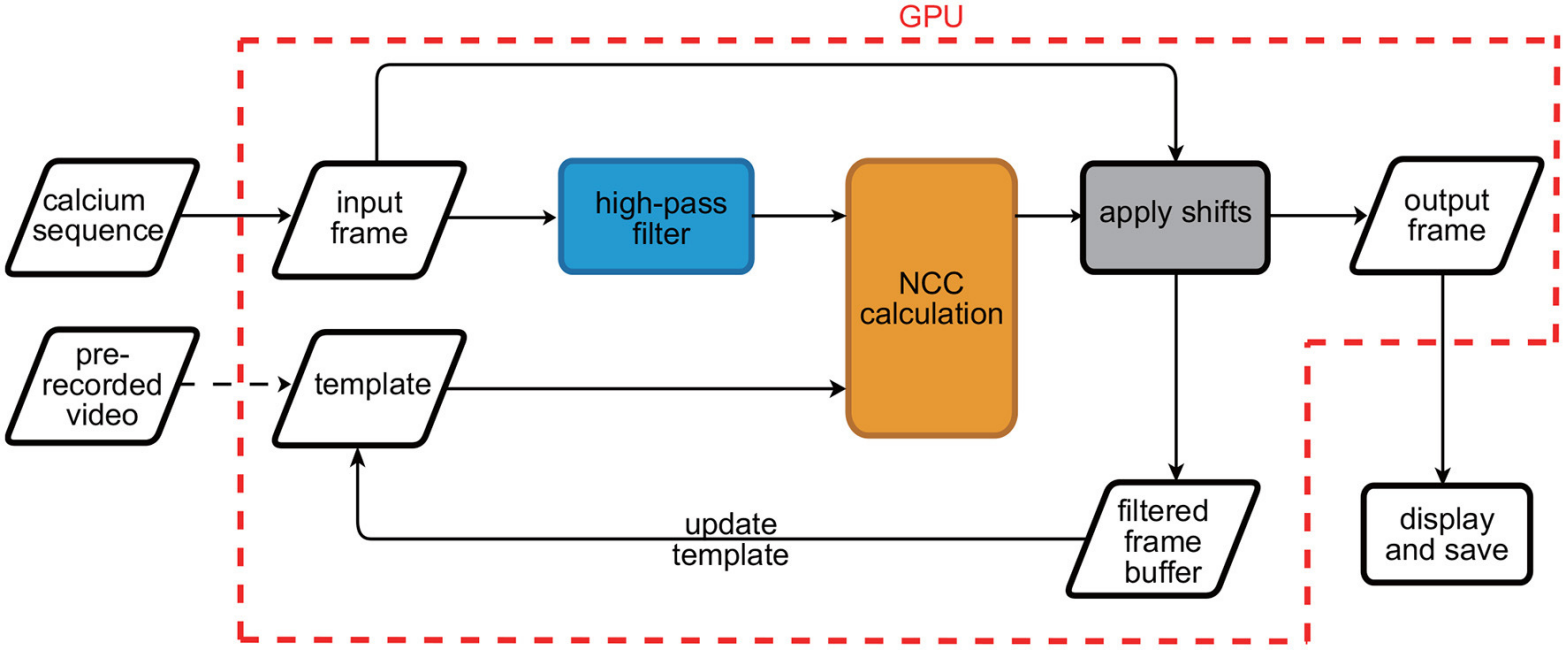
Flow chart of the RTMC algorithm. The initial template is generated based on a prerecorded video. Next, the input frame is copied and filtered. Each filtered frame is aligned with the template and translated with the raw frame. The colored procedures in the chart represent the specific processing in single frame registration. Then, the fixed filtered frame is stored in a buffer to update the template. Finally, the fixed raw frame is output, displayed and saved. The procedures in the red dotted box run on the GPU.

The workflow of the motion correction algorithm is shown in Figure 1, with the procedures in the red dotted box being GPU accelerated. Before running the RTMC algorithm, the plug-in generates a template by determining the mean image of a prerecorded video of the experimental animal. Next, each frame is continuously filtered and aligned with the template. The corrected filtered frames are stored in a buffer that can hold a maximum of k frames (e.g., *k* = 200). When the number of frames in the buffer reaches k, the template is updated by averaging the previous template and the mean value of the buffer content. As a result, the spatial features in the FOV that do not change over time, such as blood vessels, can be preserved to the maximum extent, improving the reliability of the template throughout the experiment. During the registration step, only the current frame and a subset of the previously registered frames are used. Therefore, the algorithm satisfies the online registration requirements of calcium data streams. The specific operation of the workflow is implemented by PyTorch. When applied to real-time experiments, the motion correction function is embedded in UCLA Miniscope DAQ software with Python/C API. The RTMC algorithm receives the frame sent by the hardware and outputs the corrected frames to the DAQ software for display and saving. The Python version of the algorithm and the implementation of RTMC plug-in are available at https://github.com/ChanghaoStudy/Real-time-Motion-Correction-Plug-In.

#### 2.1.1. High Pass Filter

Due to light scattering from different focal planes, the high-frequency structure of the spatial information is significantly suppressed. Therefore, we used a high-pass spatial filter (Pnevmatikakis and Giovannucci, 2017; Zhou et al., 2018) to enhance the spatial features and contrast. The kernel was generated from a Gaussian filter,


(1) $$\begin{array}{r} h\left(x,y\right)=e^{-\frac{{\left(x-x_{0}\right)}^{2}+{\left(y-y_{0}\right)}^{2}}{2l^{2}}} \end{array}$$

(2) $$\begin{array}{r} \tilde{h}\left(x,y\right)=h\left(x,y\right)-\bar{h}\left(x,y\right) \end{array}$$

where *h*(*x, y*) is a Gaussian convolution kernel used for background removal and (*x*_0_, *y*_0_) is the center of the kernel. The standard deviation of the Gaussian kernel is the width of neuron. For the data captured by the UCLA miniscope, we observed that the diameter of a neuron in the FOV is usually around 10 pixels. Based on empirical evidence, the size of the kernel should be three times the size of the neuron. Then, the kernel subtracts its spatial mean value $\bar{h}\left(x,y\right)$ to generate a new kernel $\tilde{h}\left(x,y\right)$ for filtering. In addition to removing the blurred background and increasing the image contrast, the component of the filter kernel with a positive value approximates the size of the cells, thus retaining the neuron shape and calcium fluorescence during the subsequent signal extraction.

#### 2.1.2. Normalized Cross-Correlation Calculation

To efficiently calculate the similarity between each frame and the template during frame alignment, we adopted the method proposed by Padfield (2010), which reduces the computational complexity by calculating the normalized cross-correlation (NCC) in the Fourier domain. Assume a reference image *f*_1_(*x, y*) and a moving image *f*_2_(*x*−*u, y*−*v*), with *f*_2_ shifted by (*u, v*). Then, the spatial form of the NCC can be defined as


(3) $$\begin{array}{l} NCC(u,v)=\\ \frac{\sum_{D(u,v)}\left[(f_{1}(x,y)-\bar{f_{1,u,v}})\cdot (f_{2}(x-u,y-v)-\bar{f_{2,u,v}})\right]}{\sqrt{\sum_{D(u,v)}\left(f_{1}(x,y)-\bar{f_{1,u,v}}\right)}\cdot \sqrt{\sum_{D(u,v)}{\left(f_{2}(x-u,y-v)-\bar{f_{2,u,v}}\right)}^{2}}} \end{array}$$

where *D*(*u, v*) is the overlap region of the two images and $\bar{f_{1,u,v}}$ and $\bar{f_{2,u,v}}$ represent the mean intensity of *f*_1_ and *f*_2_ in *D*(*u, v*). Since the cross-correlation of two images can be calculated in the Fourier domain by $CC(f_{1},f_{2})={ℱ}^{-1}(F_{1}\cdot F_{2}^{*})$, we can also infer the components in Equation (3) in the Fourier domain. The two images can be represented as *F*_1_ = *F*(*f*_1_) and $F_{2}^{*}=ℱ(f_{2}\prime )$, where *F*(·) represents the FFT operation, $F_{2}^{*}$ is the complex conjugate of the Fourier transform and $f_{2}\prime$ is *f*_2_ is rotated by 180°. In addition, assuming that *i*_1_ and *i*_2_ are images of ones with the same size as *f*_1_ and *f*_2_, respectively, we can define *I*_1_ = *F*(*i*_1_) and $I_{2}^{*}=ℱ(i_{2}\prime )$. Based on the results of Padfield (2011), the Fourier version of the NCC can be represented as


(4) $$\begin{array}{l} NCC(u,v)=\\ \frac{{ℱ}^{-1}(F_{1}\cdot F_{2}^{*})-\frac{{ℱ}^{-1}(F_{1}\cdot I_{2}^{*})\cdot {ℱ}^{-1}(I_{1}\cdot F_{2}^{*})}{{ℱ}^{-1}(I_{1}\cdot I_{2}^{*})}}{\sqrt{{ℱ}^{-1}(ℱ(f_{1}\cdot f_{1})\cdot I_{2}^{*})-\frac{{\left({ℱ}^{-1}(F_{1}\cdot I_{2}^{*})\right)}^{2}}{{ℱ}^{-1}(I_{1}\cdot I_{2}^{*})}}\cdot \;\sqrt{{ℱ}^{-1}(I_{1}\cdot ℱ(f_{2}^{\prime }\cdot f_{2}^{\prime }))-\frac{{\left({ℱ}^{-1}(I_{1}\cdot F_{2}^{*})\right)}^{2}}{{ℱ}^{-1}(I_{1}\cdot I_{2}^{*})}}} \end{array}$$

The motivation for using this approach is that computations in the Fourier domain have lower order complexities (*O*(*n*·*log*(*n*))) than those in the spatial domain (*O*(*n*^2^)), where *n* indicates the number of pixels in the computation. In addition, because of the normalization, NCC can be invariant to the linear gray value changes (Emmenlauer et al., 2009; Yu and Peng, 2011), which can reduce the influence of the illumination changes caused by the mice movements, out of focus brightness as well as wires poor contact at different time steps in the real-time experiments.

The displacement estimated by the NCC only has pixel-level accuracy. To further improve the accuracy, subpixel registration is necessary. The most commonly used subpixel registration methods include upsampling with different interpolation methods and curve fitting (Shimizu and Okutomi, 2002; Debella-Gilo and Kääb, 2011). Although some upsampling methods may be more accurate, the number of pixels used in the computation will be greatly increased, whereas the parabolic curve fitting method can be computed more efficiently. In parabola fitting, the matching integer pixel point (*x*_0_, *y*_0_) can be obtained through the NCC map. The subpixel location can be calculated by independently fitting a one-dimensional quadratic function based on the position with the highest correlation and its adjacent location in the NCC map: (*x*_0_ − 1, *y*_0_) and (*x*_0_ + 1, *y*_0_) in the *X*-direction and (*x*_0_, *y*_0_ − 1) and (*x*_0_, *y*_0_ + 1) in the *Y*-direction. The subpixel position can be estimated by Equations (5) and (6):


(5) $$\begin{array}{l} \text{Δ}x=\\ \;\frac{NCC(x_{0}-1,y_{0})-NCC(x_{0}+1,y_{0})}{2\times NCC(x_{0}-1,y_{0})-4\times NCC(x_{0},y_{0})+2\times NCC(x_{0}+1,y_{0})} \end{array}$$

(6) $$\begin{array}{l} \text{Δ}y=\\ \frac{NCC(x_{0},y_{0}-1)-NCC(x_{0},y_{0}+1)}{2\times NCC(x_{0},y_{0}-1)-4\times NCC(x_{0},y_{0})+2\times NCC(x_{0},y_{0}+1)} \end{array}$$

where *NCC*(*x*_0_, *y*_0_), *NCC*(*x*_0_ − 1, *y*_0_), *NCC*(*x*_0_ + 1, *y*_0_), *NCC*(*x*_0_, *y*_0_ − 1) and *NCC*(*x*_0_, *y*_0_ + 1) indicate the integer matching locations and their adjacent points in the NCC map. Finally, the offsets can be estimated by adding the detected subpixel displacements to the integer offsets.

### 2.2. Accuracy Assessment

To illustrate the performance of the RTMC registration algorithm, the rigid NoRMCorre and OpenCV template matching methods were selected for comparison. Since OpenCV template matching has previously been mainly used for two-photon data registration, we modified it to make it more suitable for single-photon registration by adding the high-pass filtering and template updating functions used in the RTMC algorithm. In addition, we skipped the step of shrinking the image to improve the accuracy and implemented the above method by Python. On the other hand, NoRMCorre has already been utilized for online processing in CaImAn (Giovannucci et al., 2019) which integrates NoRMCorre with high-pass filter for single photon data. Hence, we adopt the above integrated method in the online version of CaImAn for comparison.

#### 2.2.1. Assessment on Simulated Data

To quantitatively analyze the accuracy, we generated a simulated video by shifting a single frame 5,000 times. The artificial offset at each time step was random and within 10 pixels. Then, we investigated the impact of applying a high-pass filter on the registration precision. Next, we used OpenCV template matching, rigid NoRMCorre and RTMC to detect offsets in the simulated video and calculated the deviations for statistical analysis. In this step, we focused on evaluating how each algorithm detected random rigid translations.

#### 2.2.2. Assessment on Real Data

Although simulated data can provide the ground truth for quantitative analyses of registration performance, this data does not reflect all the characteristics of the calcium imaging data. Therefore, we also collected single-photon calcium data from the mouse motor cortex *in vivo*. We used the mean (CM) metric (Pnevmatikakis and Giovannucci, 2017; Tegtmeier et al., 2018) to quantify the motion correction performance of the different algorithms on real data. The CM metric is based on the correlation coefficients (CC) between the mean image of a video and each individual frame after motion correction. A higher CC score indicates a better match between the current frame and the template; thus, this metric can be used to compare the performance of different algorithms at the single-frame level. In addition, to eliminate the influence of different interpolation methods on the CC score when a frame is translated, all the interpolation methods used in the testing approaches were bilinear interpolations. Prior to performing the analysis, we removed the black border generated by shifting the frames during registration.

### 2.3. Speed and Memory Assessment

For real-time experiments, as the workflow of the algorithm needs to satisfy the requirements for online processing, the registration time of a single frame should not exceed the interval between two frames. We used a PC running Windows 10 64-bit with an Intel Core i7-8700 processor, 64 GB RAM and an NVIDIA GeForce RTX 2080 TI graph card to compare the processing speeds of the OpenCV template matching, rigid NoRMCorre and RTMC algorithms. To assess the long-term stability, we used the RTMC algorithm to perform real-time acquisition and motion correction on calcium imaging data from freely-moving mice while monitoring the memory usage of the software.

### 2.4. Real-Time Experimental Design

#### 2.4.1. Calcium Imaging Procedure

All the equipment and surgical procedures used for data acquisition were described in our previous study (Wang et al., 2019). In brief, the UCLA Miniscope was used to capture the data, and a 1.5 mm-diameter glass coverslip was pressed against the brain tissue through a skull hole. The imaging data were acquired at a rate of 20 frames per second with custom DAQ software.

#### 2.4.2. Comparison of Traces With and Without Applying RTMC

First, to illustrate the importance of motion correction, we manually labeled some neurons. The spatial calcium fluorescence intensity of each observed neuron was modeled as the neural spontaneous fluorescence plus the background fluorescence fluctuation (Pnevmatikakis et al., 2016). In single-photon calcium imaging data, the background can be decomposed into the global background and the local background, with the local background fluctuation more noticeably affecting the observed neuronal fluorescence (Zhou et al., 2018). Therefore, the neural calcium signal was calculated by subtracting the mean value of the region covered by the neuron from the fluorescence value of the local background, where the local background was an annular region with a radius slightly larger than the neuron. Thus, small differences in the neurons before and after registration could be compared. Furthermore, in most cases, neuroscientists use deconvolution to extract the spike activities from the calcium fluorescence traces. In our experiment, we used OASIS (Friedrich et al., 2017) to deconvolve the traces to illustrate the influence of motion correction on spike extraction.

#### 2.4.3. Comparison of Calcium Events Between Online and Offline Detection

To further demonstrate the performance of RTMC in real-time experiments, we designed a real-time neuronal calcium event detection task. When the calcium signal exceeded a specific threshold, such as 3 standard deviations from the fluorescence intensity of the target neuron, and then returned to this threshold, a calcium event was recorded (Yaksi and Friedrich, 2006; Mukamel et al., 2009). For real-time experiments, we used a prerecorded video to label the target neurons and generate the thresholds. After collecting all the data for the real-time experiment, we processed the recorded videos with MIN1PIPE and determined the number of calcium events of the labeled neurons using the same method, then compared the results with those of the real-time experiment. MIN1PIPE is an offline processing pipeline that uses a neural enhancement function to remove the background of each frame and a deconvolution function to denoise the traces before and after motion correction. As a result, MIN1PIPE can protect the calcium traces from the effects of fluctuating background fluorescence and noise. The MIN1PIPE result can be regarded as the offline ground truth. In addition, during the experiment, we counted the number of motion-corrected frames output from the GPU and computed the equivalent output frame rate. In theory, if the system has no delay and motion correction is used, the equivalent output frame rate should be equal to the acquisition frame rate of the scope.

## 3. Results

### 3.1. Accuracy Assessment of Motion Correction

#### 3.1.1. Assessment of Simulated Data

**Figure 2:**
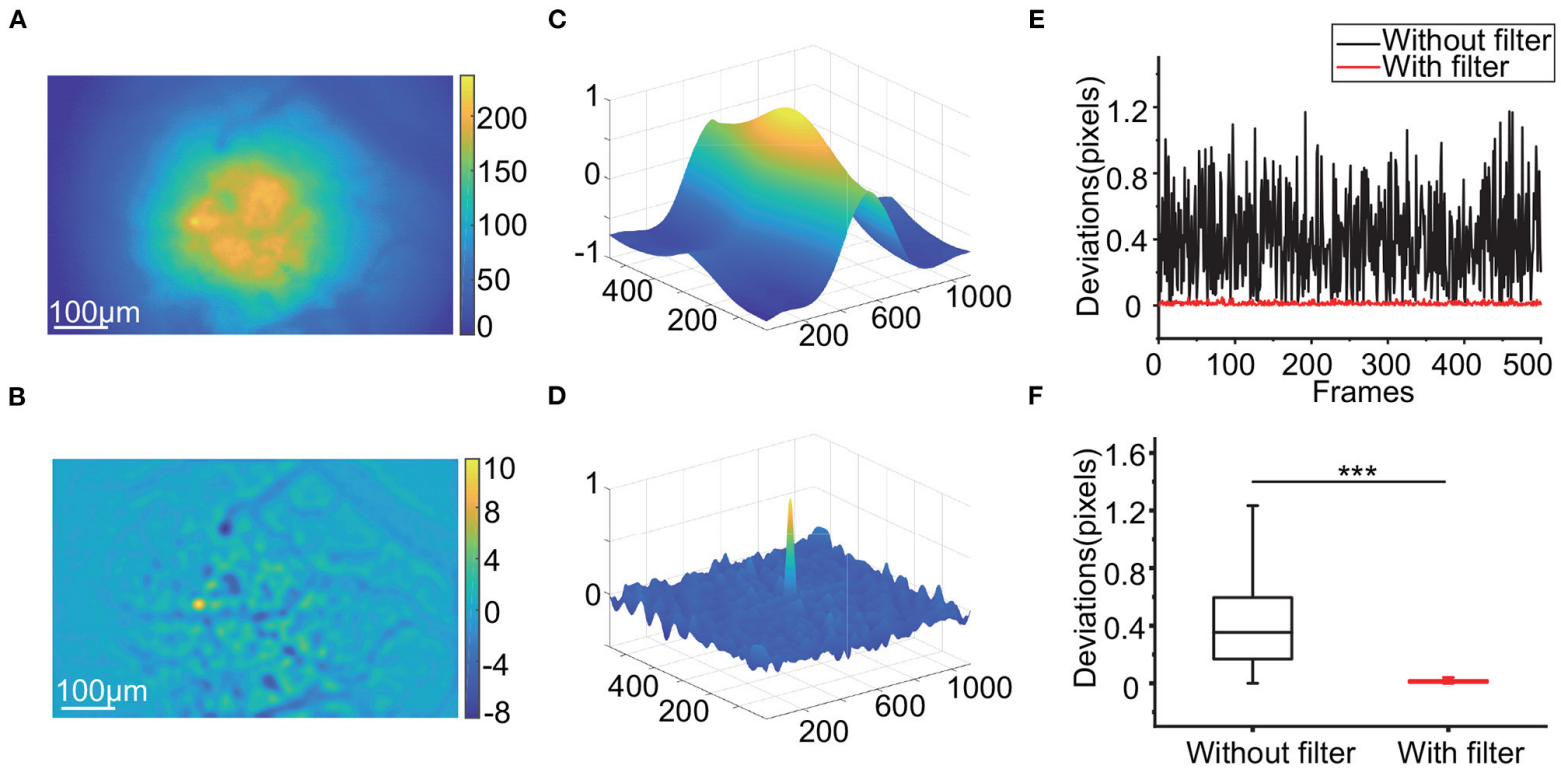
Influence of applying the high-pass filter. **(A,B)**: Raw calcium image **(A)** and filtered calcium image **(B)**. **(C,D)**: The NCC map calculated by 2 raw calcium images **(C)** and 2 filtered calcium images **(D)**. **(E,F)**: The deviations of a subset of a simulated video **(E)** and the box-plot of the deviations **(F)** with and without a high-pass filter during RTMC registration. (***p < 0.001, Wilcoxon test).

We first investigated the impact of high-pass filtering on motion correction. Light scattering from outside the focal plane blurs the whole background (Figure 2A), while high-pass filtering can produce visible textures such as mark points (Figure 2B). When calculating the similarity between each frame and the template during motion correction, the fuzzy background causes relatively high correlations in the region around the matching point in the NCC map (Figure 2C). In contrast, the NCC map has a more concentrated peak when using filtered frames (Figure 2D). After verification with the simulated data, the deviations in the frame offsets calculated after high-pass filtering can be as low as 0.1 pixels, while those calculated using raw frames for registration can reach 1.2 pixels (Figures 2E,F, *p* < 0.001, Wilcoxon test, *n* = 500 frames).

**Figure 3:**
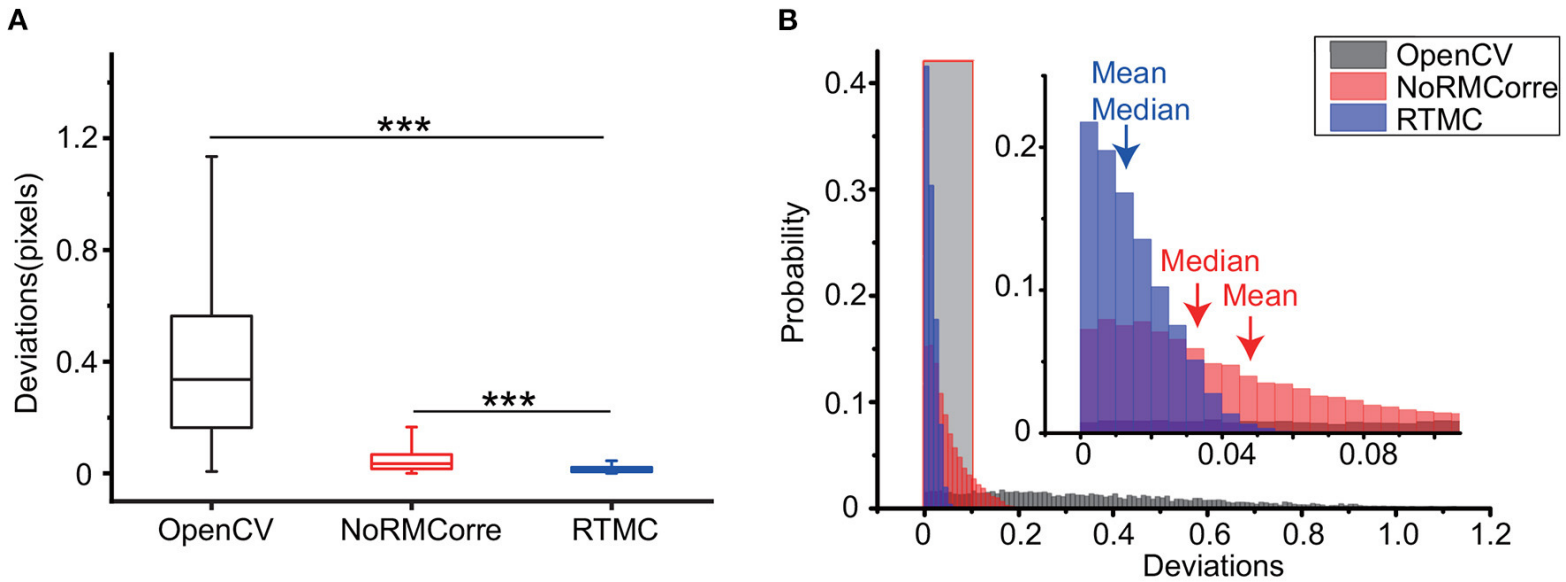
Precision comparison of the OpenCV template matching, rigid NoRMCorre and RTMC algorithms applied on a simulated video with 5,000 frames. **(A)**: Box plot of the deviations of the above 3 methods (RTMC vs. OpenCV, ***p < 0.001; RTMC vs. NoRMCorre, ***p < 0.001, Wilcoxon test). **(B)**: Histogram of the deviations. The shadowed area indicates an enlarged range from 0 to 0.1 pixels.

Next, we used the OpenCV template matching, rigid NoRMCorre and RTMC algorithms to register the simulated data to compare the precision of the three methods on single photon data. Figure 3A shows that RTMC has the smallest error and performs significantly better than the other two methods (RTMC vs. OpenCV, *p* < 0.001; RTMC vs. NoRMCorre, *p* < 0.001, Wilcoxon test, *n* = 5,000 frames). In terms of detecting offsets, RTMC produces a more accurate match between the frames and the template. The same result can be observed from the histogram of the deviations (Figure 3B). The deviations of RTMC and NoRMCorre were less than 0.2 pixels, while the error distribution of the OpenCV-based approach ranged from 0 to 1.2 pixels.

#### 3.1.2. Assessment of Mouse Motor Cortex Data

**Figure 4:**
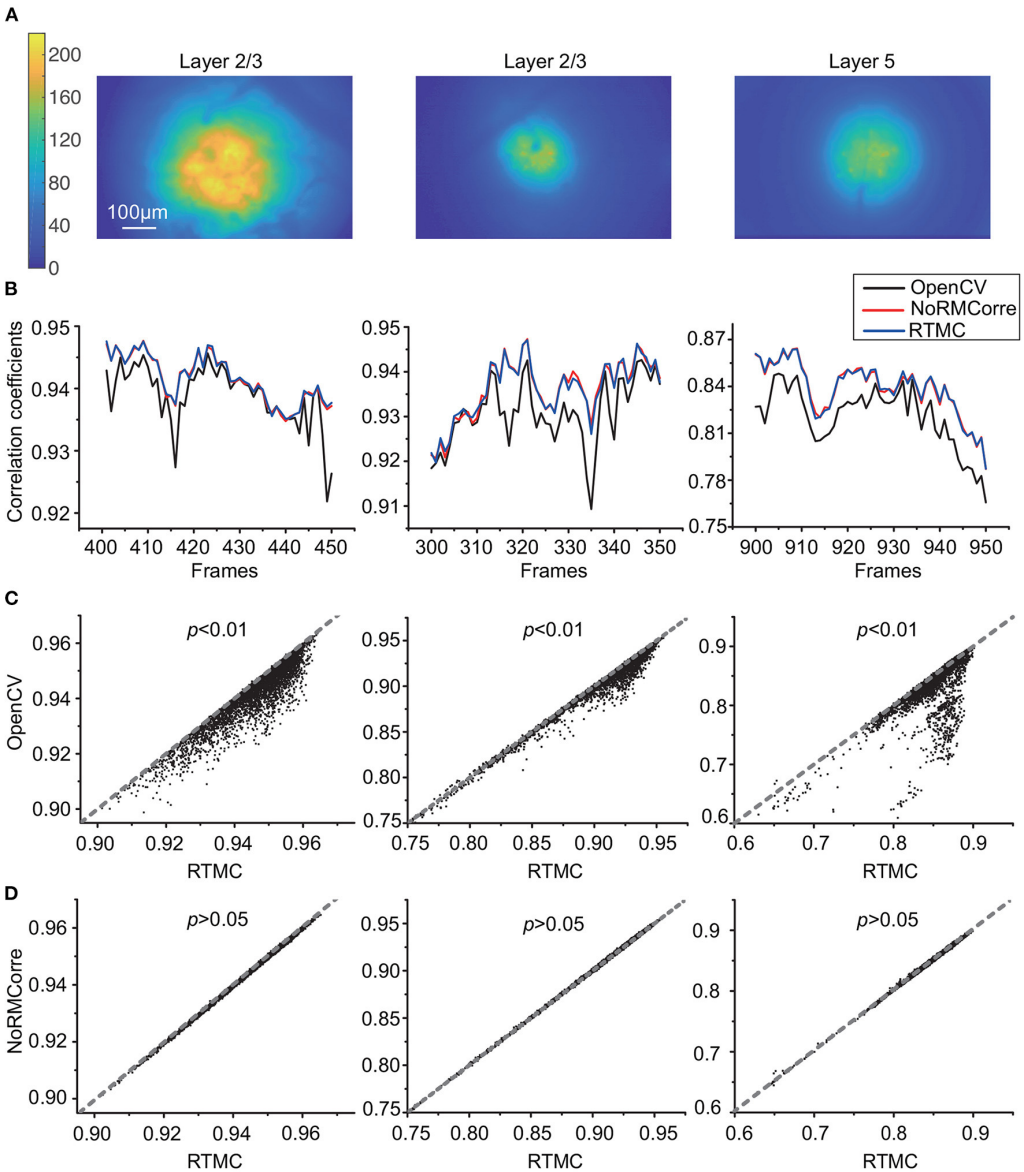
Accuracy comparison of OpenCV template matching, rigid NoRMCorre and RTMC applied to three *in vivo* mouse motor cortex datasets from layer 2/3 and layer 5, with 5,000 frames in each dataset. **(A)**: Temporal mean projection images of three datasets **(B)**: Correlation with mean (CM) metric for a subset frames in each dataset. **(C,D)**: Scatter plots of frame-by-frame CM from 3 datasets of RTMC vs. OpenCV **(C)** and RTMC vs. NoRMCorre **(D)**. RTMC has no significant difference with NoRMCorre and they both improve over OpenCV template matching (RTMC vs. OpenCV, *p* <0.01; RTMC vs. NoRMCorre, *p* >0.05, Wilcoxon test).

Since the simulated data only reconstruct the offsets in the FOV, we also needed to evaluate the accuracy of our method with real data. Figure 4 demonstrates the CM metric of the OpenCV template matching, rigid NoRMCorre and RTMC algorithms on three 5,000-frame mouse motor cortex videos from layer 2/3 and layer 5 of different animals. RTMC performs consistently well on datasets with different spatial characteristics such as blood vessels, illumination distribution, exposure intensity, etc. (Figure 4A). Figure 4B shows the CM metric for a subset of frames in each dataset; the OpenCV-based method had the lowest correlations for almost all the frames, while the other two methods had very close correlations. The scatter diagrams (Figures 4C,D) show the same trend: the correlation coefficients of RTMC were not significantly different from those of NoRMCorre (*p* > 0.05, Wilcoxon test, *n* = 5,000 frames), while both significantly exceeded those of OpenCV template matching (*p* < 0.01, Wilcoxon test, *n* = 5,000 frames) on nearly every frame. By observing the video (Supplementary Video S1) after motion correction, we found that there are still obvious motion residues in the FOV corrected by OpenCV template matching, whereas no visible motion artifacts in the videos corrected by the other two methods. In contrast to the results on the simulated data, there was no significant difference between the CM scores of RTMC and NoRMCorre. This is most likely because the pixel-shift difference between the two methods was approximately 0.2 pixels, which had little effect on the overall FOV. Moreover, fluctuations in the neuron population activity and local background fluorescence can lead to differences in the spatial features of the frames and the template. For the CM metric, the correlation between the frames and the template is more sensitive to changes in spatial features with hundreds of pixels than the difference between the 2 well-behaved methods. In addition, we also tested the effect of buffer size for updating template. We tested different buffer sizes ranging from 50 to 200 and found no difference in the accuracy of motion correction (Supplementary Figure S1). Therefore, we can set this parameter to 200, the same as the value used in CaImAn.

### 3.2. Evaluation of Speed and Memory

**Figure 5:**
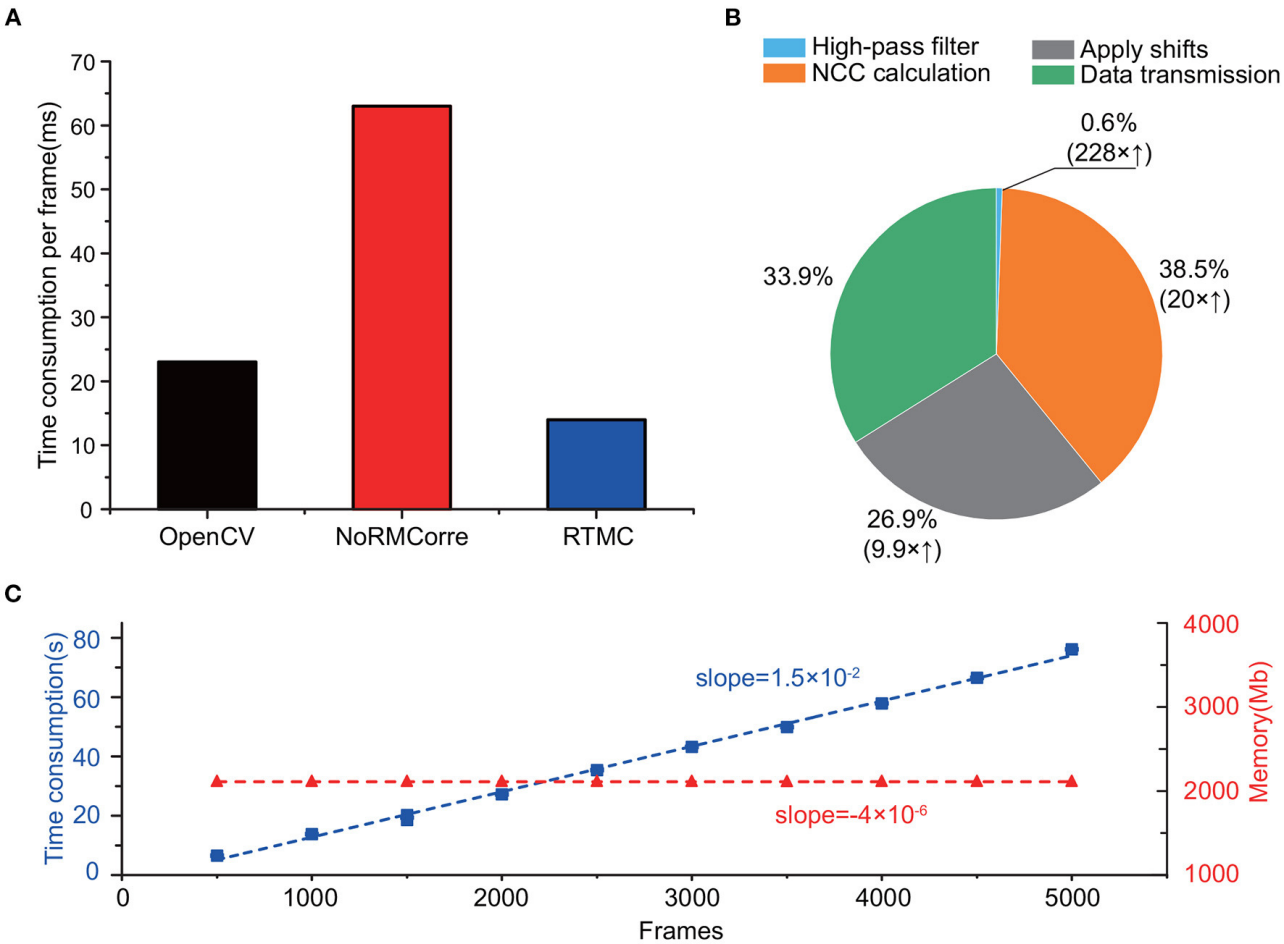
Computing speed and resources of RTMC. **(A)**: Time to process one frame with OpenCV template matching, rigid NoRMCorre and RTMC. **(B)**: The proportion of time consumed by each step while registering one frame in the RTMC workflow. The parentheses indicate the speed enhancement of the GPU over the CPU. **(C)**: Cumulative time consumption (blue, square dots) and memory occupation (red, triangle dots) of RTMC, measured per 500 frames. The dashed lines indicate the linear fit.

In addition to the satisfaction of accuracy requirements, the computational performance of the RTMC algorithm should satisfy the requirements of real-time processing. According to Figure 5A, RTMC consumes the least amount of time (15 ms) for single-frame registration, which has no delay at a frame rate of 20 Hz, while rigid NoRMCorre requires approximately 62 ms for one frame. In addition, although OpenCV template matching has a much faster rate of 23 ms per frame, its accuracy is significantly lower than that of the other two methods (Figures 3, 4). Due to GPU acceleration, the three key steps of the RTMC algorithm can be improved to varying degrees, with 228 times, 20 times and 9.9 times faster speeds achieved for high-pass filtering, the NCC calculation and applying shifts, respectively (Figure 5B). On the other hand, data transmission between the RAM and GPU adds extra processing time, which accounts for the second largest proportion (33.9%) of the process. Next, we used the RTMC algorithm to process a 5,000-frame video and counted the total processing time and memory occupation every 500 frames (Figure 5C). The cumulative time increased almost linearly at a rate of 15 ms per frame, which is consistent with the result shown in Figure 5A. Furthermore, the memory usage during the 5,000 frame registration is nearly constant (approximately 2,110 Mb), which demonstrates that high-throughput data streams can be processed by the RTMC algorithm.

### 3.3. Application to Signal Extraction With Freely-Moving Mice

#### 3.3.1. RTMC Leads to Better Calcium Trace Quality

**Figure 6:**
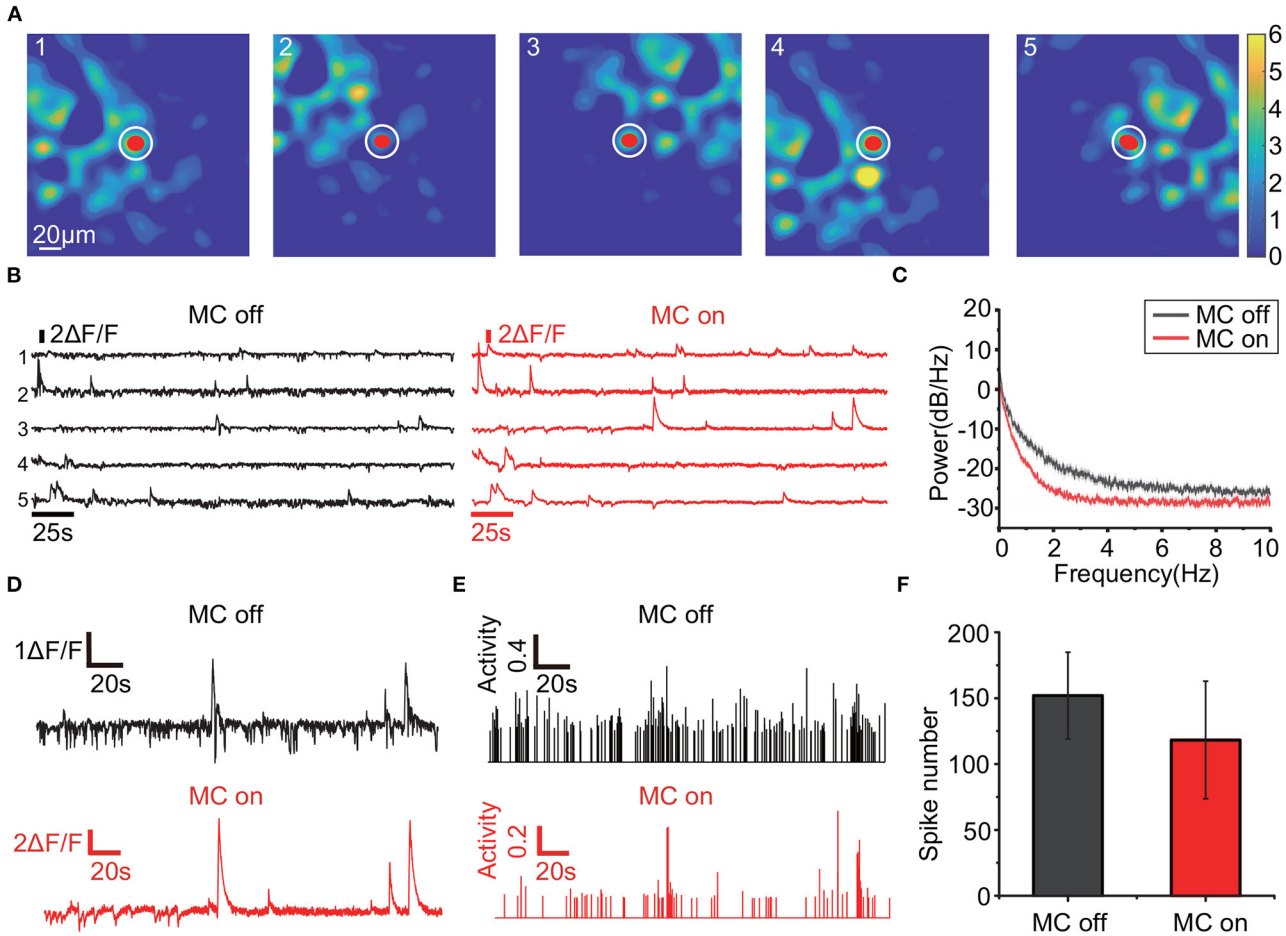
Traces comparison when RTMC is on and off. **(A)**: Positions of the 5 observed neurons (red dots) and the local background (white circles). The trace of each neuron is obtained by subtracting the mean fluorescence values of the regions covered by the neuron from the local background. **(B)**: The traces of the 5 neurons in **(A)**. The black traces were extracted without motion correction, while the red traces were extracted with motion correction using RTMC. **(C)**: Mean power spectrum of the traces with (red) and without (black) motion correction from 3 mice (15 neurons). The shaded area indicates the s.e.m. **(D)**: Enlarged example trace of the third neuron in **(A)** before and after motion correction. **(E)**: Spikes of the third neuron in **(A)** extracted by OASIS before and after motion correction. The spikes were thresholded by 3 SD of the inferred spikes activities. **(F)**: Mean number of spikes from the neurons in **(C)** extracted by OASIS. The error bar indicates the SD.

When applied to a freely-moving mouse, RTMC can improve the quality of the extracted traces (Figure 6). The calcium traces of the labeled neurons with motion correction (Figure 6A) have larger amplitudes and lower noise values than the traces extracted without motion correction (Figures 6B,D). These traces were calculated based on the mean intensities of the areas covered by the labeled ROIs, and these amplitudes are reduced when the actual neuron coverage area deviates from the labeled area. When the deviation between the two coverage regions changes frequently, the calculated fluorescence fluctuates frequently, resulting in noise. The movement-induced fluctuations in fluorescence also increase the energy of the traces, which is reflected in the power spectra and is consistent with the results of Victoria (Griffiths et al., 2020) (Figure 6C). Furthermore, since measurable calcium fluorescence is induced by neural action potentials (or spikes), the extracted traces influence the detection of action potentials (or spikes). Therefore, the transient response in the calcium traces can be evoked by the movement artifacts in the FOV, resulting in additional false positive spikes. After thresholding with 3 standard deviations of the spike activities, the inferred spikes in the motion-corrected traces are sparser and more concentrated (Figure 6E), which is consistent with the calcium dynamics model proposed in OASIS. The total number of spikes in the traces (mean±SD=118±44.6, *n*=15) was significantly less than that in the uncorrected traces (152±32.9, *n*=15), indicating that movement-induced false positive spikes were highly suppressed (Figure 6F). Therefore, the traces acquired by RTMC more accurately reflect the calcium fluorescence induced by neuronal spikes.

#### 3.3.2. Real-Time Calcium Event Detection Using RTMC in Comparison With Offline Detection Using MIN1PIPE

**Figure 7:**
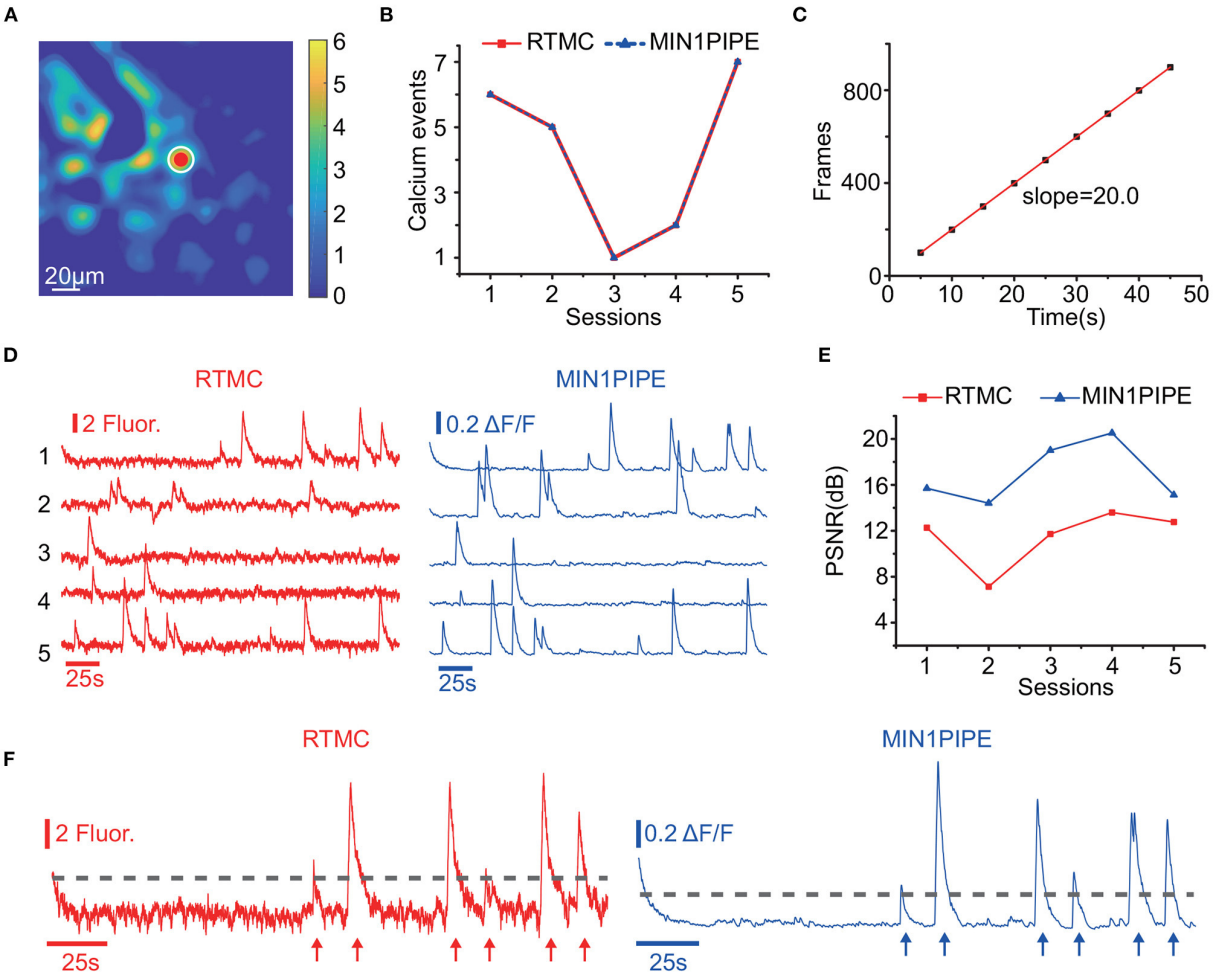
Calcium event detection in real-time with RTMC and offline with MIN1PIPE. The data were collected at 20 Hz. **(A)**: The target neuron (red dot) and local background (white circle). **(B)**: Number of detected calcium events in 5 sessions (each session lasts 250 seconds). Real-time detection was implemented by RTMC and manual labeling (red square and solid line), while offline detection was implemented by MIN1PIPE (blue triangle and dashed line). **(C)**: Cumulative number of frames received every 5 seconds while RTMC is running with motion correction enabled. The linear fit represents the equivalent frame rate. **(D)**: The traces extracted in real-time by RTMC and offline by MIN1PIPE in 5 sessions. **(E)**: The peak signal-to-noise ratio (PSNR) of the traces extracted in real-time by RTMC and offline by MIN1PIPE. **(F)**: Enlarged example of the trace of the first session in **(D)**. The dotted line indicates the threshold for calcium events, and the arrows indicate the temporal position of the events.

For real-time calcium event detection, RTMC also has high performance. Figure 7 shows the results of RTMC on a real-time calcium event detection task with a target neuron (Figure 7A). The real-time detection results were the same as those of offline detection extracted by MIN1PIPE (Figure 7B). Moreover, at a frame rate of 20 Hz with motion correction, the cumulative received frame number at each 5 s timestep was nearly equal to the theoretical number, despite the occasional frame drop (Figure 7C). After linear fitting, the growth rate of the received frame number was 20 frames per second, which is consistent with the acquisition frame rate, ensuring that RTMC can align frames during real-time data acquisition. Further analysis of these results revealed that all the traces in the 5 sessions of the 2 scenarios had similar shapes, including the peaks in each session (Figure 7D). Furthermore, the onset times of the calcium events extracted by RTMC matched the offline ground truth (Figure 7F). However, the directly extracted traces during real-time acquisition had more noise due to background fluctuations, resulting in a lower peak signal-to-noise ratio (PSNR) (Figure 7E), while MIN1PIPE dramatically improved the PSNR with its complex signal enhancement and deconvolution algorithms. In other words, although RTMC provides a real-time signal extraction approach for single-photon calcium data, motion correction is not the only factor that can influence the calcium signal quality. For example, background removal and deconvolution can improve the signal quality before and after signal extraction. To obtain higher PSNR signals, the above preprocessing and postprocessing are necessary, which is another challenge for real-time calcium signal extraction.

## 4. Discussion

In this study, we demonstrate an open-source real-time motion correction plug-in for single-photon calcium imaging data. To meet the real-time requirement, we used rigid motion correction and GPU acceleration, which had a similar or better accuracy to other rigid registration methods. In the real-time calcium event detection experiment, RTMC extracted neuron activities and achieved the same results as offline processing. Although the PSNR of the real-time extracted signals was lower than those of the offline analysis, this was due to the absence of signal enhancement and deconvolution functions rather than registration errors. Thus, RTMC can provide motion correction for signal extraction, but additional algorithms for higher signal quality need to be developed.

The OpenCV-based method of Mitani et al. performed well in two-photon calcium image registration and has been used in real-time closed-loop experiments. Because two-photon absorption is a nonlinear process, fluorophores are only excited in a diffraction-limited focal volume. Thus, the out-of-focus excitation and bleaching were significantly lower than those in one-photon data (Svoboda and Yasuda, 2006). In addition, the wavelengths of the extraction light in two-photon microscopy are in the near-IR spectrum, which has better tissue penetration than the visible light used in single-photon microscopy (Oheim et al., 2001). However, the above features do not exist in single-photon imaging data, whose spatial information is affected by light scattering. Hence, due to the difference between two-photon and single-photon calcium data, the OpenCV-based method does not account for the complexity of the data and thus cannot accurately detect displacements between frames. Because of the unique nature of single-photon imaging data, different processing methods are required. In this study, we incorporated high-pass filtering to improve the registration accuracy of single-photon data. We anticipate that our current pipeline can be further improved by using adapted filtering, adapted region/feature selection as well as morphological operations as the preprocessng steps before motion correction, which can also be accelerated and will be implemented in future work.

To a large extent, the presence of nonrigid artifacts in a frame during two-photon imaging is related to varying motion during a period of raster scanning. However, due to the different imaging methods, this type of nonrigid artifact cannot be observed in single-photon calcium data. On the other hand, although elastic brain deformation within the FOV can also lead to nonrigid artifacts, the lens is in direct contact with the brain tissue, which results in synchronous movement between the lens and the tissue and thus reduces nonrigid motion in the FOV to some degree. Finally, the most obvious motion artifacts in single-photon data are translations. When the lens is firmly anchored to the micro camera, other transformations, such as rotations and shears, are relatively rare. Some researches (Gauthier et al., 2018; Bollimunta et al., 2021) also adopted translation correction methods to process their single photon data before the experiments or analyses. Therefore, rigid translation correction can provide satisfactory results in most cases. In the future, we will integrate fast and accurate nonrigid registration algorithms into the software to handle more complex deformation and satisfy the demands of real-time processing.

RTMC, an open-source plug-in for capturing single-photon calcium imaging videos without movement artifacts in the FOV, has a wide range of applications in real-time closed-loop experiments. By manually labeling neurons and extracting their signals in real time, researchers can target specific neurons in experimental animals and extract their activities in response to specific behaviors. For example, RTMC can be used in brain-computer interface experiments to extract neural activities in real time and provide feedback to the experimental object (Clancy et al., 2014). Currently, at a capture frame rate of 20 Hz, the RTMC algorithm only takes approximately 15 ms; thus, we can perform more operations between two frames. For example, RTMC can be used together with online deconvolution algorithms, such as OASIS, to infer spikes from fluorescent calcium signals of the target neurons in real time and to denoise the fluorescent signals, providing higher quality signals for real-time experiments. RTMC can also be used as a preprocess step of real-time automatic neuron extraction algorithm, such as ONACID-E (Friedrich et al., 2021). We look forward to ONACID-E to be implemented on GPU in order to achieve real-time automatic extraction of neuron signals from single-photon calcium imaging data together with RTMC, thus providing a platform for closed-loop experiments based on single-photon calcium imaging.

## Data Availability Statement

The raw data supporting the conclusions of this article will be made available upon reasonable request.

## Ethics Statement

The animal study was reviewed and approved by the Animal Care Committee of Zhejiang University, China.

## Author Contributions

ML, LF, and SZ conceived the project. ML drafted the manuscript. ML and CL performed the algorithm and software development. XC, HJ, and HY provided technical support for software design. All authors contributed to manuscript revision and approved the submitted version.

## Funding

This research was supported by the National Key Research and Development Program of China (2017YFE0195500), Key Research and Development Program of Zhejiang Province (2021C03003, 2021C03107, and 2022C03029), and Zhejiang Lab (2021KI0PI02).

## Conflict of Interest

The authors declare that the research was conducted in the absence of any commercial or financial relationships that could be construed as a potential conflict of interest.

## Publisher's Note

All claims expressed in this article are solely those of the authors and do not necessarily represent those of their affiliated organizations, or those of the publisher, the editors and the reviewers. Any product that may be evaluated in this article, or claim that may be made by its manufacturer, is not guaranteed or endorsed by the publisher.
