# Supplementary material for: An Open-Source Real-Time Motion Correction Plug-In for Single-Photon Calcium Imaging of Head-Mounted Microscopy
Source: Front Neural Circuits. 2022 Jun 24;16:891825. doi: 10.3389/fncir.2022.891825 (PMC9265215; doi:10.3389/fncir.2022.891825)
Supplement: Supplementary file 1 [file Data_Sheet_1.PDF]

Supplementary Material

1 SUPPLEMENTARY FIGURES

1.1 Figures

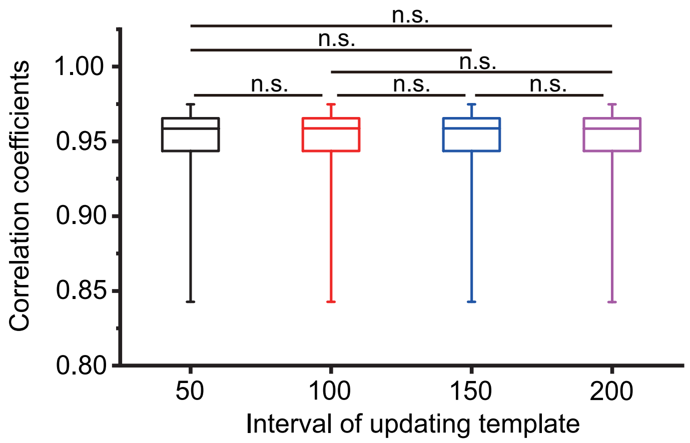

Figure S1. Boxplot of the correlation with mean (CM) metric of the real data after motion correction by RTMC. The videos are processed with different buffer sizes for updating template from 50 to 200. The results have no significant difference (p>0.05, Wilcoxon test).
